# 

**Published:** 1886-06

**Authors:** 


					﻿National Dental Association.
The next regular bi-ennial meeting of the National Dental
Association of the United States of America will be held at
Washington, D. C., July 27, 28, and 29, 1886.
OFFICERS.
President, R. B. Winder, Baltimore, Md.
First Vice-President, John B. Rich, New York.
Second “	“	V. E. Turner, Raleigh, N. C.
Third “	“	W. W. Ford, Macon, Ga.
Fourth “	“	E. Parmly Brown, Flushing, N. Y.
Fifth “	“	J. H. Coyle, Thomasville, Ga.
Secretary, R. Finley Hunt, Washington, D. C.
Treasurer, H. B. Noble, Washington, D. C.
Very respectfully,
R. Finley Hunt,
Secretary, N. D. A., U. S. A.
Board of Examiners.
There will be a regular meeting of the Minnesota State Board
of Dental Examiners, July 25th, at St. Paul (immediately after
the session of the State Dental Society), for the purpose of ex-
amining applicants to practice in the State of Minnesota.
J. H. Martindale, Secretary.
pHE pENTAL pEQI^TER,
A Monthly Magazine Devoted to the Interests of the
DENTAL PROFESSION.
Terms Reduced to $2.00 Per Tear. Single Copies, 25 Cents.
EDITED BY PROF. J. TAFT, D.D.S.
Published by SAM A, CROCKER I CO., Ohio'Dental ad Surgical Depot
The volume commences in January and closes in December.
The Register, being issued monthly is always fresh, therefore Indispensable
to the practitioner who desires to keep posted up with the new inventions and
improvements which are daily developed. Neither expense nor effort will be
spared to give value and variety to its contents Articles will be illustrated with
well executed engravings whenever they will add to the interest or assist to ex-
planation.
Its extensive circulation and frequency of issue make it one of the BEST DEN*
TAL ADVERTISING MEDIUMS in the United States.
All subscriptions must begin with January or July.
Local or special notices, five lines or less, will be inserted for $3 each insertion ;
over five lines, 50 cts. per line.
All advertising bills payable in advance, or at furthest, after the issue of the
first number containing the advertisement. All transient advertisements to be
paid for in advance.
^CONTRIBUTIONS SOLICITED.
Exclusively Editorial Communications should be addressed to the Editor.
The latest number of the Kegistrr may be ordered with or without other goods
at any time, and all letters should be addressed to
SAM’L A. CROCKER & CO., Ohio Dental and Surgical Depot, Cincinnati, 0.
				

